# Correction: Wimalawansa, S.J. Physiological Basis for Using Vitamin D to Improve Health. *Biomedicines* 2023, *11*, 1542

**DOI:** 10.3390/biomedicines12122807

**Published:** 2024-12-11

**Authors:** Sunil J. Wimalawansa

**Affiliations:** Medicine, Endocrinology & Nutrition, Cardio Metabolic Institute, (Former) Rutgers University, North Brunswick, NJ 08901, USA; suniljw@hotmail.com

The publication (Wimalawansa 2023, [1]) has been amended to increase the clarity of citation sources within the text. In addition, in the original publication, a few errors from the edited proof were not incorporated into the final version, thus these were corrected here. The authors state that the scientific conclusions are unaffected. This correction was approved by the Academic Editor. The original publication has also been updated.

## Text Corrections

Text corrections to clarify citation sources and revised text have been made in the following sections:

Abstract;

1. Introduction;

1.1. Vitamin D_3_ (Cholecalciferol);

1.2. Fundamental Benefits of Vitamin D;

1.3. Vitamin D Metabolites and Analogs and Their Clinical Uses;

1.4. Consequences of Hypovitaminosis D;

1.5. Intracellular Synthesis of Calcitriol and Benefit from Vitamin D;

1.6. Why This Study Is Important and Necessary;

2. Vitamin D Generation—Genomic and Non-Genomic Actions;

2.1. Genomic Actions of Calcitriol;

2.2. Non-Genomic Actions of Calcitriol;

3. Clinical and Randomized Control Studies (RCTs);

3.1. Contributions from Recent Clinical Studies;

3.2. Reasons for Failure of Recent Vitamin D Randomized Controlled Clinical Studies;

3.3. Other Critical Aspects to Consider, Specifically for Vitamin D-Related RCTs;

3.4. Examples of Larger Vitamin D Interventional RCTs with Significant Study Design Errors;

3.5. Contrasts between Negative and Positive RCTs;

3.6. What to Expect from RCTs;

3.7. Systems and Different Tissues May Need Different Serum Concentrations of 25(OH)D;

4. Pharmacodynamics and the Underlying Mechanisms of Calcitriol;

4.1. Autocrine and Paracrine Signaling;

4.2. Optimum Concentrations of Circulating 25(OH)D Concentrations;

4.3. Importance of Raising Population 25(OH)D Concentrations to Reduce Morbidities;

4.4. Factors That Modify the Functions of CYPP450 Enzymes and VDRs;

4.5. The Importance of Prescribing the Proper Type and Doses of Vitamin D;

4.6. Vitamin D Doses to Maintain Therapeutic Serum 25(OH)D Concentrations;

4.7. Vitamin D Dose Recommendations;

5. Conclusions.

## Modifications in Figure and Table

In Table 1, the following edits have been made to improve clarity.

**Table 1:** Key nutrient clinical study recruitment criteria: Clinical studies should be conducted to test the hypothesis that intervention (e.g., vitamin D supplements) benefit recipient subjects.

The Goal	Action Needed
Recruit only vitamin D-deficient subjects	Measurement of baseline serum 25(OH)D concentration. Recruit subjects with 25(OH)D serum levels less than 20 ng/mL (50 nmol/L) for clinical studies and RCTs.
Sufficient sample size	Based on statistical power calculation (on the effect size and the standard error of the mean).
Sufficient doses and the right frequency of administration (e.g., daily or weekly—not monthly or semi-annually)	Avoid administration of vitamin D at a frequency or less than once in two weeks. Age-appropriate proper doses must be used. Use the appropriate vitamin D dose to raise serum 25(OH)D concentration to a sufficient (target) level to achieve the intended outcome.
Ensure the sufficiency of co-nutrients and co-factors	For optimal function, supplemented nutrients interact and act synergistically with other nutrients. In the case of vitamin D (or calcium), ensure the availability of co-factors and supporting elements, such as magnesium, vitamin K_2_, etc.
Ensure the desired blood concentration is achieved (e.g., 25(OH)D concentration)	In longer trials, serum 25(OH)D concentrations should be measured after initiation of the intervention (e.g., approximately in four months).
Sufficient duration of the study	Shorter trials (e.g., acute infections) that last a few weeks vs. longer trials. Whereas chronic diseases, such as metabolic disorders, cancer, and osteoporosis, require several years of follow-up.
Keep the study clean	Study subjects should not take additional doses of index nutrients, including multivitamins, which might provide more of the same nutrients. Importantly, nutrients like vitamin D should not piggyback on pharmaceutical trials.
Keep the study simple	Use a simpler (uncomplicated) protocol with fewer variables and minimum number of study groups necessary to test the hypothesis. This decreases the number of subjects needed, improves statistical power, and makes for more straightforward interpretations and meaningful conclusions.
Clinical and statistical meaningfulness	Clinical study protocol must test a hypothesis based on a clinically meaningful increase in the indexed nutrient in circulation [i.e., 25(OH)D]—achieving and maintaining the blood levels above the minimum target is essential.
Balanced randomizations	Minimize confounders.
Target serum 25(OH)D concentration (and the therapeutic window)	Supplementation should bring serum 25(OH)D levels to a sufficiency level (at least above 40 ng/mL; 100 nmol/L) (in the case of infections, cancer, and autoimmune diseases, above 50 ng/mL)—the target goal of the clinical study.
Maintain a sustained effect	Maintain circulatory 25(OH)D concentrations above the target level during the entire study period in the interventional group [106].
Have firm-hard endpoints	The protocol should define hard primary endpoint(s)—e.g., complications such as reduced fractures, number needed to treat (NNT) to save one life, hospitalizations, ICU admissions, or deaths.
Over-the-counter nutrients, especially the index nutrient (e.g., vitamin D), and supplements, and vitamins should not be allowed to be taken during clinical studies [105,107,108]	Allowing patients to consume micro-nutrients from such a source will reduce the difference between the groups (the effect size) and the statistical power.
Statistical analyses	Correlation should be made with serum 25(OH)D concentrations achieved (active vs. control group) after supplements consumption [or at least, the changes (∆) from the baseline], but not with the administered dose.

Figure 4A has been updated for clarity. We added a purple broken blue line on the upper right corner to represent those with rare indications (i.e., resistant to standard therapies) mentioned in Sections 3.7 and 4.7, such as drug-resistant migraine and cluster headaches, psoriasis, asthma, etc. These groups of patients need the maintenance of significantly higher serum 25(OH)D concentrations to achieve vital benefits between 80 and 150 ng/mL levels without demonstrable adverse effects. Patients/subjects must be on very low calcium diets and managed under close medical supervision by experts in this field. Hypercalcemia generally would not manifest under 150 ng/mL.

**Figure 4:**
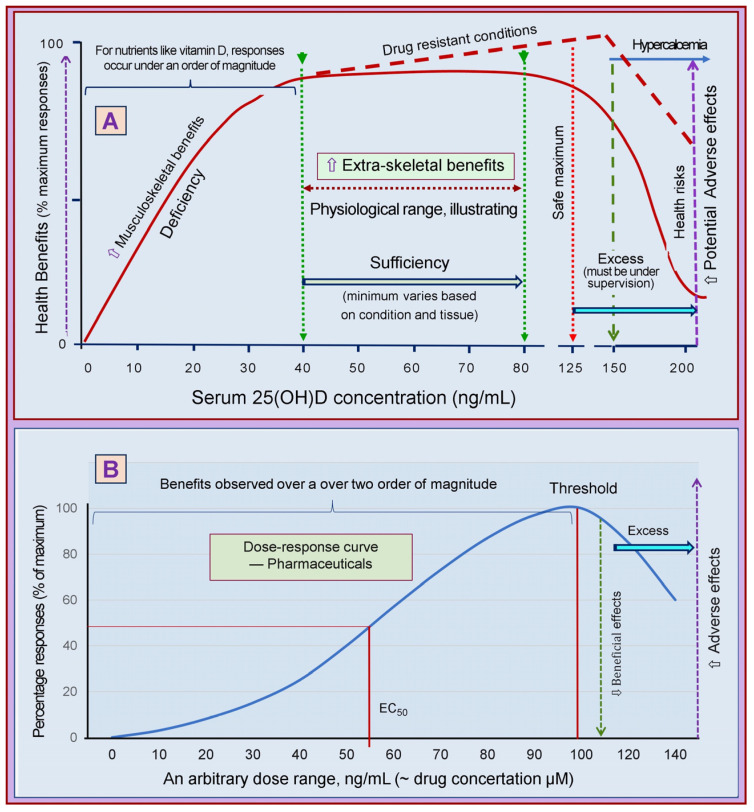
Illustrates pharmacodynamic differences and dose-response curves between nutrients and pharmaceutical agents. While nutrients show an abrupt response, dose-response curves of pharmaceutical agents spread over a broader range. (**A**) Depicts an example of vitamin D and dose responses. Providing more would not have additional physiological benefits when it reaches sufficiency for a given tissue/system. Furthermore, the response range is narrow, about half an order of magnitude. (**B**) The response range expands with pharmaceutical agents over an order of magnitude, and the response curve is shallow. The broken red line illustrates that the beneficial effects of vitamin D could continue without causing hypercalcemia when high doses are administered with very low calcium intakes and under close medical supervision.

## Citation Corrections

In the original publication [1], 11 references [22,28,29,31,55,72,122,132,133,151,169] were not cited.

22. Nogues, X.; Ovejero, D.; Pineda-Moncusí, M.; Bouillon, R.; Arenas, D.; Pascual, J.; Ribes, A.; Guerri-Fernandez, R.; Villar-Garcia, J.; Rial, A.; et al. Calcifediol Treatment and COVID-19-Related Outcomes. *J. Clin. Endocrinol. Metab.* **2021**, *106*, e4017–e4027. https://doi.org/10.1210/clinem/dgab405.
28. Ter Braake, A.D.; Shanahan, C.M.; De Baaij, J.H. Magnesium Counteracts Vascular Calcification: Passive Interference or Active Modulation? *Arterioscler. Thromb. Vasc. Biol.* **2017**, *37*, 1431–1445.
29. Wimalawansa, S.J.; Dissanayake, C.B. Factors Affecting the Environmentally Induced, Chronic Kidney Disease of Unknown Aetiology in Dry Zonal Regions in Tropical Countries—Novel Findings. *Environments* **2019**, *7*, 2.
31. Jamilian, M.; Amirani, E.; Asemi, Z. The effects of vitamin D and probiotic co-supplementation on glucose homeostasis, inflammation, oxidative stress and pregnancy outcomes in gestational diabetes: A randomized, double-blind, placebo-controlled trial. *Clin. Nutr.* **2019**, *38*, 2098–2105.
55. Yan, Y.; Gong, Z.; Xu, Z. Vitamin D supplementation and colorectal cancer prognosis. *Med. Oncol.* **2019**, *36*, 69.
72. Munasinghe, L.L.; Willows, N.D.; Yuan, Y.; Ekwaru, J.P.; Veugelers, P.J. Vitamin D Sufficiency of Canadian Children Did Not Improve Following the 2010 Revision of the Dietary Guidelines That Recommend Higher Intake of Vitamin D: An Analysis of the Canadian Health Measures Survey. *Nutrients* **2017**, *9*, 945.
122. Murai, I.H.; Fernandes, A.L.; Sales, L.P.; Pinto, A.J.; Goessler, K.F.; Duran, C.S.C.; Silva, C.B.R.; Franco, A.S.; Macedo, M.B.; Dalmolin, H.H.H.; et al. Effect of a single high dose of vitamin D3 on hospital length of stay in patients with moderate to severe COVID-19: A randomized clinical trial. *JAMA* **2021**, *325*, 1053–1060. https://doi.org/10.1001/jama.2020.26848.
132. Fernandez, G.J.; Ramírez-Mejía, J.M.; Castillo, J.A.; Urcuqui-Inchima, S. Vitamin D modulates expression of antimicrobial peptides and proinflammatory cytokines to restrict Zika virus infection in macrophages. *Int. Immunopharmacol.* **2023**, *119*, 110232.
133. Amado, C.A.; García-Unzueta, M.T.; Fariñas, M.C.; Santos, F.; Ortiz, M.; Muñoz-Cacho, P.; Amado, J.A. Vitamin D nutritional status and vitamin D regulated antimicrobial peptides in serum and pleural fluid of patients with infectious and noninfectious pleural effusions. *BMC Pulm. Med.* **2016**, *16*, 99.
151. Gunville, C.F.; Mourani, P.M.; Ginde, A.A. The role of vitamin D in prevention and treatment of infection. *Inflamm. Allergy Drug Targets* **2013**, *12*, 239–245. https://doi.org/10.2174/18715281113129990046.
169. Wimalawansa, S.J. Vitamin D and cardiovascular diseases: Causality. *J. Steroid Biochem. Mol. Biol.* **2018**, *175*, 29–43. https://doi.org/10.1016/j.jsbmb.2016.12.016.

And removed one reference [42]. With this correction, the order of some references has been adjusted accordingly.

42. Carlberg, C. Molecular Approaches for Optimizing Vitamin D Supplementation. *Vitam. Horm.* **2016**, *100*, 255–271.
